# Isolation, identification, and pathogenicity of *Pseudoalteromonas aliena* associated with oyster disease outbreaks in summer

**DOI:** 10.1128/spectrum.00173-25

**Published:** 2025-06-23

**Authors:** Shuyi Mu, Jiejie Sun, Lei Gao, Yinan Li, Zihan Wang, Renle Chang, Jun Jiang, Liyan Wang, Lingling Wang, Linsheng Song

**Affiliations:** 1 Liaoning Key Laboratory of Marine Animal Immunology & Disease Control, Dalian Ocean University 26480, Dalian, China; 2 Liaoning Key Laboratory of Marine Animal Immunology, Dalian Ocean University 26480, Dalian, China; 3 Laboratory of Marine Fisheries Science and Food Production Processes, Qingdao National Laboratory for Marine Science and Technology 474988 , Qingdao, China; 4 Dalian Key Laboratory of Aquatic Animal Disease Control, Dalian Ocean University 26480, Dalian, China; Commonwealth Scientific and Industrial Research Organisation, Brisbane, Australia; East China Normal University, Shanghai, China; Shandong Agricultural University, Shandong, China

**Keywords:** *Crassostrea gigas*, *Pseudoalteromonas aliena*, pathogenicity, 16S rRNA, inflammation

## Abstract

With the continuous expansion of oyster farming scale, disease has become one of the main obstacles to restricting the development of oyster farming. In the present study, 20 bacterial strains were identified from *Crassostrea gigas* with pustulosis, among which *Pseudoalteromonas aliena* emerged as the predominant strain, characterized by its rod-shaped morphology and possession of flagella. *P. aliena* exhibited α-hemolytic activity at 28°C and displayed high susceptibility to all 20 chemotherapeutic agents tested. After *P. aliena* infection, the oyster mortality rate increased. The gills were swollen and eroded, and the mantle was green with pustules after *P. aliena* infection. The gill filaments exhibited swelling and necrotic cells, and the mantle showed a loose histological structure with cavities and disruption of epithelial cells. The extracellular products (ECPs) from *P. aliena* had urease, protease, and amylase activities. The potential virulence proteins identified from ECPs were GroL, ClpB, and HtpG proteins. After injection with ECPs, there was an increase in the oyster mortality rate, and the observed symptoms in gill filaments and mantle were consistent with those observed after *P. aliena* infection. In addition, the mRNA expressions of inflammation- and programmed cell death-related genes were significantly upregulated in gills and mantle. The relative abundances of *Vibrio*, *Arcobacter,* and *Pseudoalteromonas* also exhibited a significant increase in the gills and mantle. The results demonstrated that *P. aliena* was the pathogenic bacterium for oysters, and its pathogenicity mechanism was systematically clarified, which provided valuable insights for the prevention and control of bacterial disease in oysters.

**IMPORTANCE:**

Disease has currently emerged as one of the principal impediments to restricting the development of the oyster breeding industry. In the present study, *Pseudoalteromonas aliena* was identified from *Crassostrea gigas* with pustulosis. After *P. aliena* infection, the oyster mortality rate increased. The gills were swollen and eroded, and the mantle was green with pustules. Extracellular products (ECPs) from *P. aliena* had urease, protease, and amylase activities. The potential virulence proteins identified from ECPs were GroL, ClpB, and HtpG proteins. After injection with ECPs, the oyster mortality rate increased. The mRNA expressions of inflammation- and programmed cell death-related genes in gills and mantle increased significantly, and the relative abundances of *Vibrio*, *Arcobacter,* and *Pseudoalteromonas* exhibited a significant increase after *P. aliena* infection. The results demonstrated that *P. aliena* was the pathogenic bacterium for oysters, and its pathogenicity mechanism was systematically clarified, which provided valuable insights for the prevention and control of bacterial disease in oysters.

## INTRODUCTION

Oyster is one of the most important aquatic shellfish farming species in the world and has important economic value (1). However, bacterial infections frequently occur among oysters due to factors such as water quality and breeding density. Moreover, these bacterial diseases often exhibit explosive outbreaks that result in a rapid increase in mortality rates within cultured organisms, leading to severe economic losses (2–4).

There exist numerous pathogenic bacteria in bivalves, and the majority of them are classified as *Vibrio* (5). Several *Vibrio* species have been isolated from cultured diseased bivalves, including *Vibrio aestuarianus* (6), *Vibrio coralliilyticus*, *Vibrio splendidus* (7), *Vibrio tapetis*, *Vibrio tasmaniensis*, and *Vibrio mediterranei* (8). Vibriosis is generally considered to be an opportunistic disease that affects the host or its larvae (9). *Vibrio* can induce pathogenesis by means of extracellular products (ECPs), such as lipopolysaccharide, exotoxin, hemolysin, and protease (10). The pathogen abundance of the *Pseudoalteromonas* genus was relatively high in the lesions of oysters with pustules, suggesting that it might be a potential pathogen of oysters.

*Pseudoalteromona*s, which is widely distributed in marine environments, has the capability to secrete bioactive compounds, including bacteriostatic, algolytic, bactericidal, and cellulose-degrading agents (11, 12). It is present abundantly in the microbial consortia inhabiting oil-polluted water bodies (13–15). Only a few reports have identified *Pseudoalteromonas* species as pathogenic to marine organisms, such as fish (sea bream and European sea bass) (16, 17), blue swimming crab (18), juvenile Pacific abalone (19), and alga (20). The “standstill disease” abalone mortality rates were high when exposed to *Pseudoalteromonas shioyasakiensis* at 28°C. This opportunistic pathogen possessed an exceptionally strong growth capacity in the mucus of abalone feet and undermined the first mucosal immune barrier of the feet within 3 days.

The cultivation of oysters occupies a prominent position within China’s aquaculture industry, as the country boasts the largest oyster farming area and highest production output globally (21). The occurrence of elevated mass mortalities of the Pacific oysters has been observed in northern China in recent years, particularly during summer (22). In the present study, *Pseudoalteromonas aliena,* as the most dominant among the culturable bacteria isolated from lesions of diseased oysters, was further characterized with the objectives to (i) investigate its physiological and biochemical characteristics, (ii) determine its pathogenicity, and (iii) explore underlying pathogenic mechanisms in oysters, thereby establishing a solid foundation for epidemiological investigations and the development of targeted drugs against oyster diseases.

## MATERIALS AND METHODS

### Experimental animals

The diseased and healthy oysters were both collected from a local farm in Dalian, Liaoning Province, China. The diseased oysters displayed a vibrant green coloration, along with the presence of pustules in the mantle, swelling of the gills, and extensive erosion. The healthy oysters were fed with spirulina every 2 days and kept for 7 days for the following experiments.

### Bacteria isolation and identification

The diseased oysters were repeatedly rinsed with sterile seawater. The affected areas were then excised, ground, and inoculated onto 2216E agar medium for cultivation at 28°C for 24 h. Based on colony morphology, the bacterial strain isolated from the plates was then sub-cultured on 2216E liquid medium to obtain pure colonies. The bacterial strain was stored at −80°C in 30% glycerol.

The protocols for isolating DNA from individual colonies and performing PCR amplification were referenced in the previous study (23). The primer synthesis and sequencing of the PCR amplification products were performed by Sangon Biotech (Shanghai) Co., Ltd. The acquired 16S rRNA sequence of the bacterium was subjected to a BLAST search using the EzBioCloud (EzBioCloud.net | Search for Bacteria or Archaea) and National Center for Biotechnology Information database. The phylogenetic trees were constructed using the neighbor-joining methods in MEGA 11 software, based on the 16S rRNA sequences of *P. aliena* and 10 other species belonging to the genus *Pseudoalteromonas*.

### Phenotypic analysis of *P. aliena*

*P. aliena* was cultured in 2216E liquid medium at 28°C for 24 h, and the bacterial culture was centrifuged at 3,500 × *g* for 5 min. The pellet was resuspended and washed twice using sterile seawater to eliminate interference from the medium. The morphological structure of *P. aliena* was observed under a transmission electron microscope (TEM). Growth was assessed on 2216E liquid medium at temperatures ranging from 28°C after incubation for 24 h. The physiological and biochemical characteristics of *P. aliena* were tested by a physiological and biochemical tube (Qingdao Haibo, China).

### Growth determination

A single colony was randomly picked and then inoculated in 2216E liquid medium and cultured until the OD_600_ value reached 0.5 at 28°C. Later, 100 µL of the bacterial solution was inoculated into 5 mL of 2216E liquid medium and cultured at 28°C for 24 h. OD_600_ value was measured every 2 h.

### Drug sensitivity test of *P. aliena*

The susceptibility of *P. aliena* to antibiotics was determined using a disk-diffusion method, employing commercially available antibiotic disks (Hangzhou Microbial Reagent Co., Ltd., China). A total of 20 common antibiotics (Amoxicillin, Penicillin, Cefotaxime, Cefoperazone, Florfenicol, Chloramphenicol, Erythromycin, Neomycin, Kanamycin, Streptomycin, Tetracycline, Doxycycline, Rifampin, SXT, Achromycin, Sulfisoxazole, Enrofloxacin, Norfloxacin, Azithromycin, and Roxithromycin) were applied as dots on 2216E agar medium that had been uniformly coated with 100 µL suspensions of *P. aliena*. The plates were incubated at 28°C for 24 h to observe the presence of a zone around the antibiotic spots. The judgment criteria are defined as follows: a zone of inhibition with a diameter ≤12 mm is classified as low sensitivity or non-sensitive, a diameter between >13 mm and ≤17 mm indicates moderate sensitivity, and a diameter >18 mm is categorized as high sensitivity.

### Hemolytic activity of *P. aliena*

The hemolytic activity of *P. aliena* was assessed using sheep blood agar (2216E agar medium supplemented with 5% sheep blood). *P. aliena* culture was inoculated as 50 µL droplets onto the surface of the sheep blood agar and incubated at different temperatures (28°C and 37°C) for a duration of 1 week. The presence of a distinct zone on the blood agar was defined as positive hemolysis (24). The hemolytic circle around the drop of *P. aliena* was analyzed both by observation with the naked eye and measurement of the diameter.

### *P*. *aliena* challenge

The isolated bacteria *P. aliena* was separately cultured in 2216E at 28°C for 24 h and harvested by centrifugation at 3,500 × *g* for 5 min. Then, the bacteria *P. aliena* was washed twice and serially diluted to 1 × 10^8^ CFU mL^−1^ with sterile seawater. Following a 7-day period of administering 40 healthy oysters, they were divided into two groups (20 oysters per group), namely, blank and *P. aliena* groups. There were three replicates in each group. In the immersion experiment, *P. aliena* was added to seawater at final concentrations of 1 × 10^5^ CFU mL^−1^. The oysters in the *P. aliena* group and control group were cultured at 25°C. Over the following nine days, the mortality was recorded every day.

According to Koch’s postulates, the bacteria were reisolated from the gaping or dying oysters with an immersion challenge. The gills and mantle were homogenized under aseptic conditions and cultured on 2216E agar medium to isolate and purify bacteria as described above. The bacterial colonies inoculated on 2216E agar medium were harvested for DNA extraction and 16S rRNA sequencing.

### Histopathologic analysis of gills and mantle from infected oysters

Samples of moribund oysters from the experimental challenged groups were fixed with formaldehyde fixative for 24 h. The lesions in the gills and mantle were dissected from infected oysters. After embedding in paraffin, the sections were cut with a microtome (Leica, Germany). The samples (5 µm) were first deparaffinized and rehydrated, then stained with a commercial kit of hematoxylin and eosin (H&E) or modified Brown & Brenn Gram stain (Beyotime, China) for histopathologic and bacterial examination, respectively.

### ECPs characterization

The ECPs were generated using the cellophane overlay method as previously described (25). Briefly, bacteria were cultured in 5 mL of 2216E liquid medium at 28°C for 24 h. Subsequently, a volume of 1 mL of exponential phase culture (OD_600_ = 1) was spread onto sterile cellophane film placed overlying agar plates containing 2216E medium. Following incubation at 28°C for durations of 12, 24, 36, 48, and 60 h, the cells were detached from the cellophane by washing with PBS (0.01 M, pH = 7) using a volume of 2 mL and subsequently removed through centrifugation at a speed of 14,000 × *g* for 20 min at 4°C. The resulting supernatant was filtered sequentially through membranes with pore sizes of 0.45 and 0.22 µm before being concentrated via lyophilization. Its concentration was determined utilizing the protein-dye binding principle (26). Finally, ECPs were stored at −80°C.

Protein quantitation was performed using the Bradford Protein Assay Kit (Beyotime, China). The 12.5% sodium dodecyl sulfate polyacrylamide gel electrophoresis (12.5% SDS-PAGE) was used to separate the secreted ECPs. Then, the peptide sequences of the differentially expressed proteins were analyzed by using a combination of gas-phase ion/ion chemistry and tandem mass spectrometry (Applied Biosystems, China).

### Determination of ECP enzymes

After 12 h of cultivation, the *P. aliena* strain with the highest extracellular enzyme content was selected, and its extracellular enzymatic activities, including phospholipase, lipase, amylase, and urease, were quantitatively assessed. The 2216E agar plates added with 0.2% starch, 8% fat-free milk, 0.2% urea, and 0.8% Tween-80 were prepared accordingly. Twenty microliters of each bacterial suspension were spot-inoculated onto the corresponding Petri dishes and incubated at 28°C for 24 h. The test was performed in triplicate.

### ECPs toxicity assay

The ECPs of bacteria cultured at 28°C were collected at 12 h, as the ECP concentration at 12 h was the highest. The oysters were separated into five groups (40 oysters per group). There were three replicates in each group. Oysters in each group were cultured at 25°C and received an injection with 100 µL ECPs at concentrations of 8 × 10^2^, 8 × 10^1^, and 8 × 10^0^ µg mL^−1^ and sterile seawater. In the blank control group, the oysters receiving an injection with sterile seawater were cultured at 16°C. The oysters were observed twice daily to record the mortality.

### RNA extraction and reverse transcription quantitative PCR analysis

The total RNAs were extracted from hemolymph using TRIzol reagent (Invitrogen) according to the protocols. Subsequently, cDNA was performed using the TransScript One-Step gDNA Removal and cDNA Synthesis SuperMix Kit (TransGen) and diluted to a 1:20 ratio for reverse transcription quantitative PCR (RT-qPCR). The mRNA expression levels of inflammation- and programmed cell death-related genes were detected with their corresponding primers (Table 1) by SYBR Green fluorescent RT-qPCR in a QuantStudio 6 Flex Real-time PCR System (Applied Biosystems). The fragments of elongation factor (*Cg*EF) were used as an internal control. The relative expression levels of target genes were calculated using the 2^−ΔΔCT^ method (27).

**TABLE 1:** Sequences of the primers used in this study

Primer	Sequence (5′−3′)
RT-qPCR primers	
*Cg*EF-RT-F	AGTCACCAAGGCTGCACAGAAAG
*Cg*EF-RT-R	TCCGACGTA TTTCTTTGCGA TGT
*Cg*Ferritin-RT-F	AGAAACCCGACCGTGATGAG
*Cg*Ferritin-RT-R	CTGTGCATCCTGGTGACTGT
*Cg*GPX4-RT-F	AAAGTATGCTGAGGAGAAGGGGCT
*Cg*GPX4-RT-R	CTTTTCACTGGCTTCCCTTCTTTG
*Cg*SLC40A1-RT-F	GCTGTTCCTCATCAGCCTGT
*Cg*SLC40A1-RT-R	AACTGCAGCACACAGTACGA
*Cg*DLAT-RT-F	TTGTAGTCACACCTGGGGCAGAGTA
*Cg*DLAT-RT-R	ACCGCTTGGCTATTGTCTTTCTCAT
*Cg*FDX1-RT-F	ATCAAAGACAAGCCCACAGACGA
*Cg*FDX1-RT-R	CTTGGTAACAATCACTTGACAGCCTAAT
*Cg*SLC3A1-RT-F	GTCGACGTCAACACACCACT
*Cg*SLC3A1-RT-R	CGTGAAACTCACGTCGTCAC
*Cg*ATG5-RT-F	GGAGGCTGACTCACTGAAGC
*Cg*ATG5-RT-R	CAGAGATTGGTGGTGCCCTT
*Cg*LC3-RT-F	AGGAACAGCAGCTACCTATGC
*Cg*LC3-RT-R	TGGAGTTCAGTGACATTCGGTT
*Cg*P62-RT-F	TGCAGAATGTTGGCCAGAGT
*Cg*P62-RT-R	GCATTTTCTGCGCTTGTGGA
*Cg*Caspase8-2-RT-F	GTGGTGCCTAAAGGACAACT
*Cg*Caspase8-2-RT-R	TCAACACTTTCAGTAAAACATCAA
*Cg*Caspase-3-RT-F	GGCTGACTTTCTGATTGCTT
*Cg*Caspase-3-RT-R	ATGTCGGAGTGGGAGGTGTT
*Cg*AIF1-RT-F	GGGAAGACCAAGACCCAC
*Cg*AIF1-RT-R	AGGAAGACTATCCAATGC
*Cg*C3-RT-F	AAGCAGCCCAACATACGTCA
*Cg*C3-RT-R	TACGCGTGCTCTACGTCATC
*Cg*HMGB1-RT-F	AAGGGAAAAGACCCCAACGAT
*Cg*HMGB1-RT-R	GCACCACCGCCTCCCTTT
*Cg*IL17-1- RT-F	GCGAACGCCACAGTGTCAAA
*Cg*IL17-1- RT-R	GACGCTACGAGGAAATACGGAC
*Cg*IL17-5- RT-F	TCTGGCTGACTCTCGTCCTTG
*Cg*IL17-5- RT-R	GACCCTGTCGTTGTCCTCTACC

### DNA extraction, high-throughput sequencing, bioinformatic analysis, and function prediction

Nine oysters were randomly sampled from each group to analyze the hemolymph microbiota, and there were three groups. The genomic DNAs of samples were extracted using the E.Z.N.A. Stool DNA Kit (Omega, USA) according to the manufacturer’s instructions. The following steps were performed according to the previous study (28).

### Statistical analysis

All data were analyzed using the SPSS 22.0 software package and expressed as mean ± S.D. (*N* = 3). Differences were considered significant at *P* < 0.05 (*), highly significant at *P* < 0.01 (**), and extremely significant at *P* < 0.001 (***).

## RESULTS

### The identification and characterization of *P. aliena*

**Fig 1:**
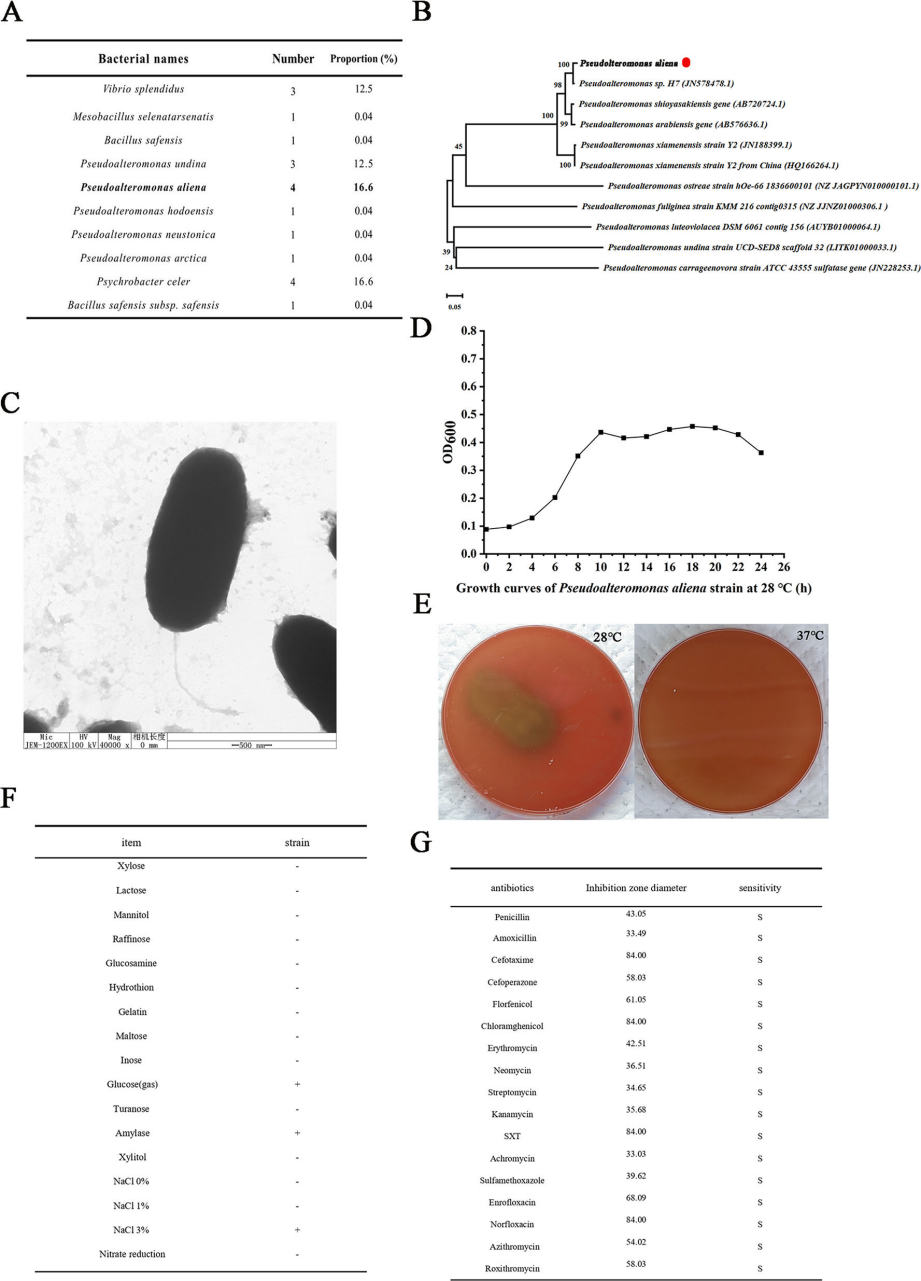
Identification, isolation, and physiological and biochemical characteristics of *P. aliena*. (**A**) The bacterial strains were isolated from diseased oysters. (**B**) The phylogenetic tree was constructed using the nucleotide sequence of *P. aliena* and those from the other 10 *Pseudoalteromonas* species. The scale bar corresponds to 0.05 substitutions per site. (**C**) The morphological characteristics of *P. aliena* were observed by TEM. (**D**) The growth curve of *P. aliena* under the cultivation conditions at 28°C. (**E**) The hemolysis assay of *P. aliena* cultured at 28°C and 37°C for 72 h. (**F**) Physiological and biochemical indices of *P. aliena*. Note: “+,” positive; “−,” negative. (**G**) Drug sensitivity of *P. aliena*. Note: R, resistant; I, weak sensitive; S, sensitive. A value represents the mean ± S.D. (*N* = 3).

The diseased oysters for bacterial isolation displayed a vibrant green coloration, along with the presence of pustules in the mantle, swelling of the gills, and extensive erosion. A total of 20 bacterial strains were isolated from the lesions of moribund oysters. A total of 20 stains were identified through 16S rRNA sequencing. Among which, *P. aliena* held the highest proportion (Fig. 1A). So, it was selected as the subject for subsequent experimental investigations.

The phylogenetic tree was constructed with the utilization of 16S rRNA data, encompassing 11 bacterial species from the *Pseudoalteromonas* genus. Strain *P. aliena* was clustered together with *Pseudoalteromonas* sp*. H7* (Fig. 1B). *P. aliena* exhibited a rod-shaped morphology with approximately 1 µm in length. Single polar flagellum was observed with a length of about 4.5 µm. Negative staining electron microscopy revealed rod-shaped bacteria distinguished by a single flagellum located at one end (Fig. 1C). *P. aliena* entered an initial logarithmic growth phase at 28°C after 2 h and attained a stationary phase after 10 h (Fig. 1D). After incubation at 28°C for 72 h, a pronounced α-hemolytic zone was observed surrounding *P. aliena* colonies on sheep blood agar, while no hemolysis was observed at 37°C (Fig. 1E). In the biochemical identification assay of *P. aliena*, positive reactions were observed for glucose, amylase, and 30% NaCl, whereas all other tested biochemical assays showed negative results (Fig. 1F). According to the antibiotic susceptibility assay, *P. aliena* exhibited high sensitivity to all 17 chemotherapeutic agents (Fig. 1G).

### The challenge assay of oysters with *P. aliena* and the histopathological analysis

**Fig 2:**
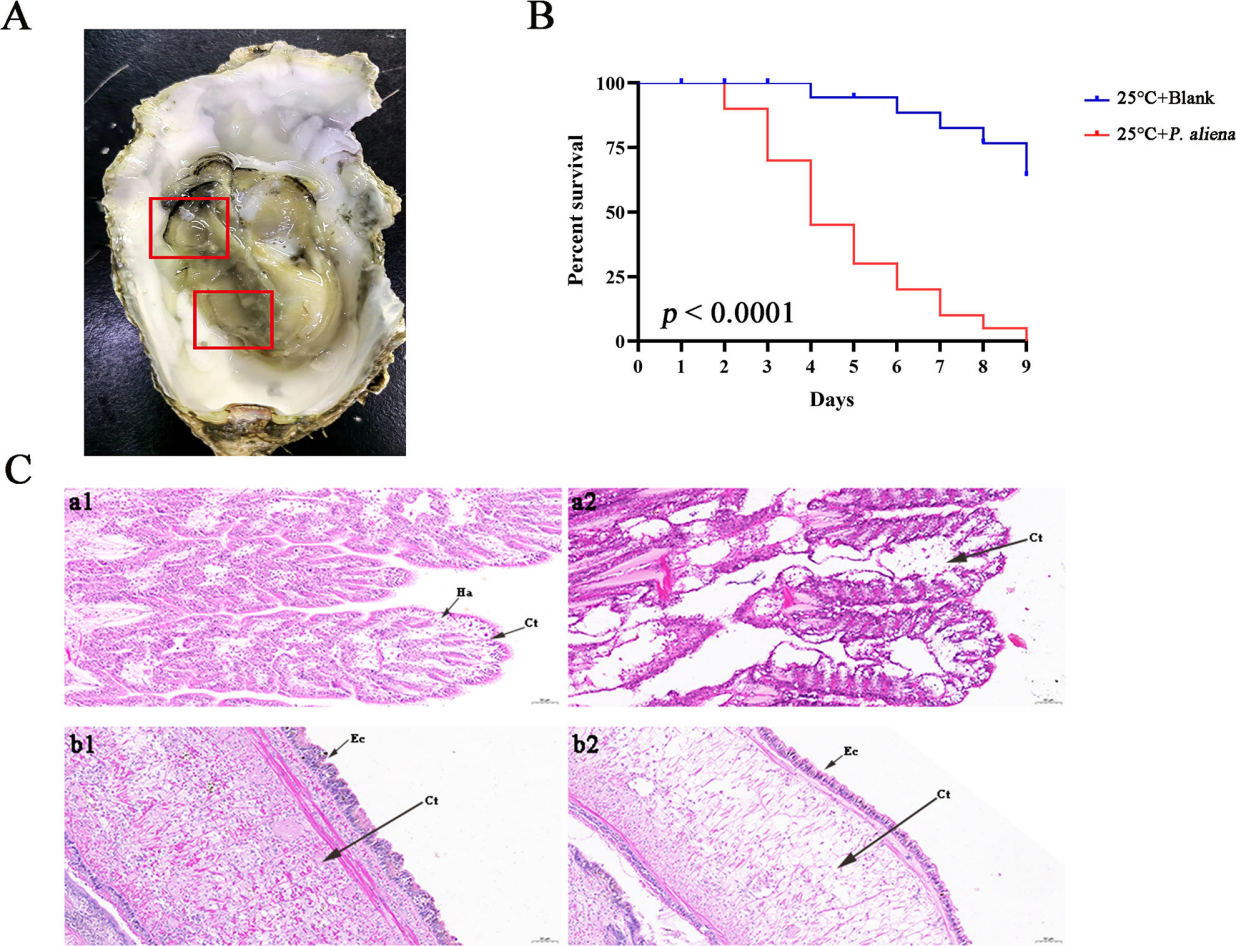
The appearance, mortality rate, and tissue sections of oysters after *P. aliena* infection. (**A**) The symptoms of oyster after immersion experiment with *P. aliena*. (**B**) Survival curves of oyster infected with *P. aliena* at 25°C. X axis, days. Y axis, oyster survival rate. The survival rate was recorded every day, *n* = 20. (**C**) H&E-stained histological sections of tissues in the immersion experiment. a, Gills; b, mantle; 1, untreated group; 2, infection group; Ha, hemocyte; Ct, connective tissue; Ec, epithelial cell.

**Fig 3:**
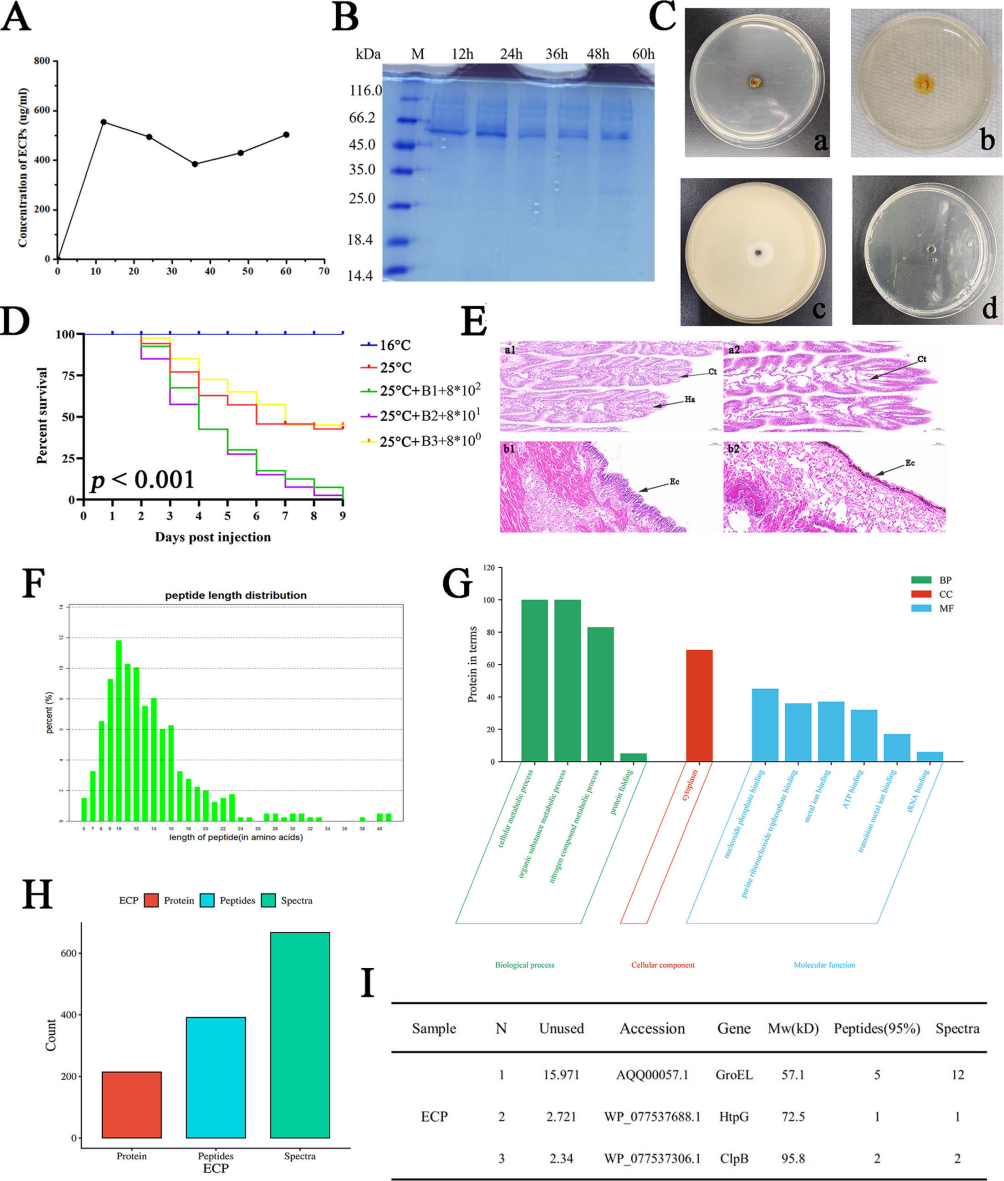
Extraction and analysis of ECPs from *P. aliena*, as well as the oyster mortality rate and tissue sections after ECP stimulation. (**A**) Concentration of ECPs under different culture times. (**B**) The SDS-PAGE analysis of ECPs. (**C**) The enzyme activity assay of ECPs. a, Amylase detection plate; b, urease detection plate; c, proteinase detection plate; d, lipase detection plate. (**D**) Cumulative survival rate of oysters after ECPs stimulation. X axis, days post-infection. Y axis, oyster survival rate. The survival rate was recorded every day for 9 days, *n* = 40. (**E**) Histological sections of oysters after ECPs stimulation. a, Gills; b, mantle; 1, untreated group; 2, ECPs stimulation group; Ha, hemocyte; Ct, connective tissue; Ec, epithelial cell. (**F**) Peptide mass deviation distribution of ECPs. (**G**) The statistics of ECPs by GO analysis. (**H**) The total protein of ECPs. (**I**) Pathogenesis-related proteins in ECPs.

The initial mortality event was recorded on the second day post-challenge in the immersion experiment at 25°C, with a bacterial concentration of 1 × 10^5^ CFU mL^−1^. After *P. aliena* infection, the oysters exhibited symptoms consistent with those observed in diseased oysters from the local farm (Fig. 2A). The mortality rate in the *P. aliena* group reached 100% at 9 days, whereas it remained at 40% in the blank group (Fig. 2B). In accordance with Koch’s postulates, the reisolated bacterial strain was confirmed to exhibit identical 16S rRNA fragment sequences and phenotypic characteristics (white colonies on 2216E agar) as those of *P. aliena*. The gills were swollen and eroded, and the mantle was green with pustules after *P. aliena* infection. The gill filaments exhibited swelling and necrotic cells, and the mantle showed a loose histological structure with cavities and disruption of epithelial cells (Fig. 2C). After ECPs stimulation, the gills and mantle also presented the same symptoms and histopathological alterations as those of the oysters infected with *P. aliena* (Fig. 3E).

### The pathogenicity of ECPs

The ECPs from *P. aliena* were harvested at 28°C after 12, 24, 36, 48, and 60 h of culturing. The total protein content of the ECPs at these time points was measured as follows: 554.1, 493.8, 384.4, 429.3, and 503.1 µg mL^−1^. The ECP concentration exhibited a peak at 12 h and reached a minimum at 36 h (Fig. 3A). Subsequently, there were various proteins ranging from 25 to 116 kDa in ECPs (Fig. 3B). The ECP components from *P. aliena* strain formed hydrolytic zones on the protease, amylase, and urease plates, while no visible changes were observed on the lipase plate (Fig. 3C). The pathogenicity was confirmed after ECP-treated oysters at 25°C. On the ninth day following the injection of ECPs, the mortality rates of oysters were 100%, 100%, and 55%, respectively. And their corresponding concentrations were 8 × 10², 8 × 10¹, and 8 × 10⁰ µg mL^−1^, while the mortality rate of the control group was 50% with seawater at 25°C (Fig. 3D). There was no death in the blank group with seawater at 16°C.

The mass deviation of the identified peptides in all samples was quantified, revealing that the distribution of each peptide’s mass deviation fell within the range of ±20 ppm (Fig. 3F), indicating excellent accuracy in mass spectrometry detection. The sample was subjected to mass spectrometry detection, and there were 214 protein profiles identified with 391 peptide segments among them, and the number of secondary mass spectra corresponding to the proteins was 667 (Fig. 3H). Virulence proteins with high confidence levels (CV ≥95%), including GroL, ClpB, and HtpG proteins, were identified (Fig. 3I). These proteins were primarily related to dehydratase activity, tRNA binding, unfolded protein binding, ATP binding, and metal ion binding. Furthermore, these proteins were predominantly localized in the cytoplasm (Fig. 3G).

### The mRNA expression levels of inflammatory programmed cell death-related genes in gills and mantle after *P. aliena* infection

**Fig 4:**
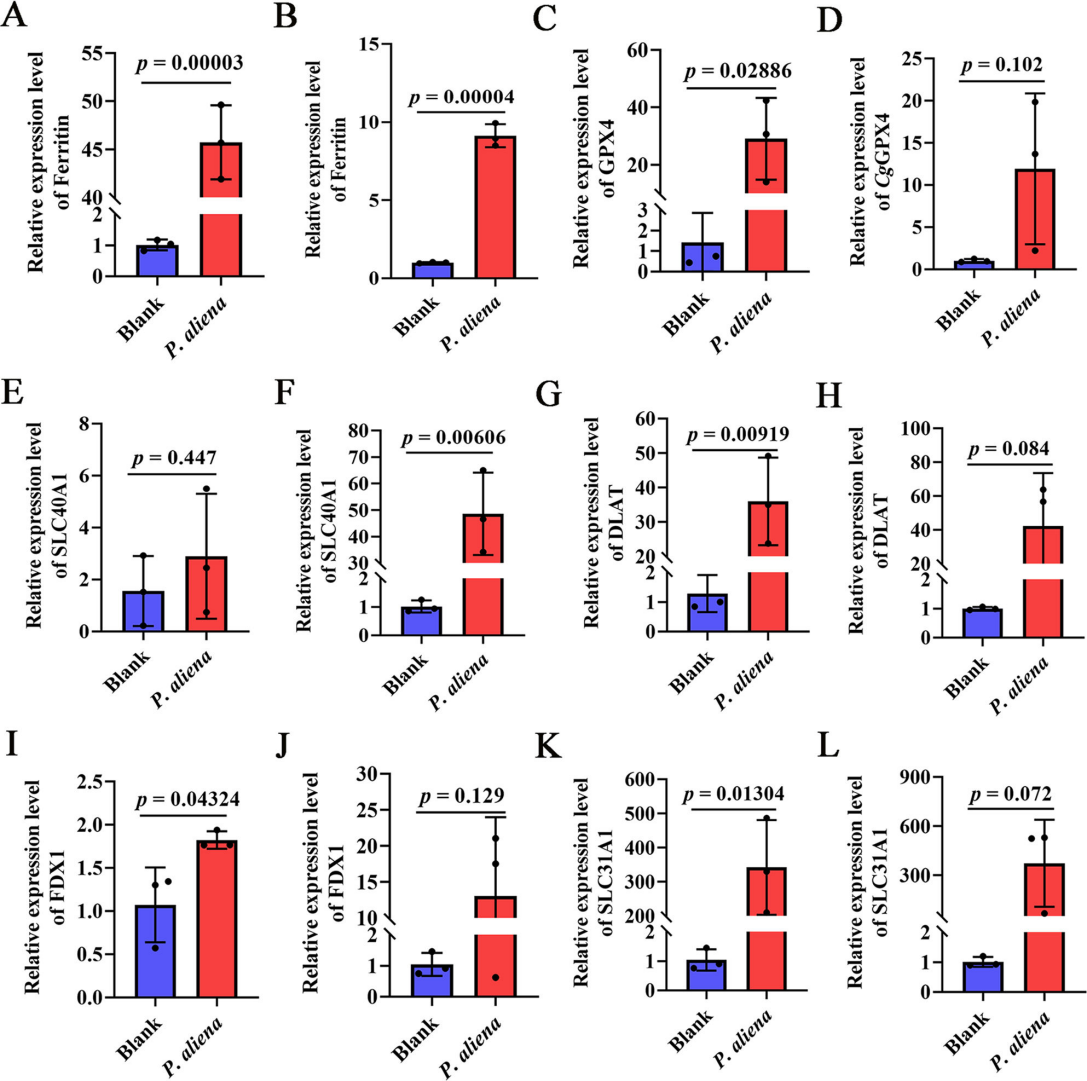
The relative mRNA expression levels of non-inflammatory programmed cell death-related genes in gills and mantle after *P. aliena* infection. (A, C, E, G, I, and K) The mRNA expression levels of *Cg*Ferritin, *Cg*GPX4, *Cg*SLC40A1, *Cg*DLAT, *Cg*FDX1, and *Cg*SLC31A1 in gills. (B, D, F, H, J, and L) The mRNA expression levels of *Cg*Ferritin, *Cg*GPX4, *Cg*SLC40A1, *Cg*DLAT, *Cg*FDX1, and *Cg*SLC31A1 in the mantle. *P. aliena*: *P-1*. Error bars represent the mean ± S.D. (*N* = 3).

The mRNA expression levels of inflammatory programmed cell death-related genes in gills and mantle were significantly increased after *P. aliena* infection. The mRNA expression levels of *Cg*Ferritin (Fig. 4A and B), *Cg*GPX4 (Fig. 4C), *Cg*SLC40A1 (Fig. 4F), *Cg*DLAT (Fig. 4G and H), *Cg*FDX1 (Fig. 4I), and *Cg*SLC31A1 (Fig. 4K) were significantly increased after *P. aliena* infection, which were 45.28-fold, 9.12-fold, 29.05-fold, 47.93-fold, 28.06-fold, 42.3-fold, 1.69-fold, and 329.27-fold (*P* < 0.05) compared with those in the control group, respectively. There was no significant change in the mRNA expression levels of *Cg*SLC40A1 in the gills (Fig. 4E), *Cg*FDX1 in the mantle (Fig. 4J), and *Cg*SLC31A1 in the mantle (Fig. 4L).

### The mRNA expression levels of non-inflammatory programmed cell death-related genes in gills and mantle after *P. aliena* infection

**Fig 5:**
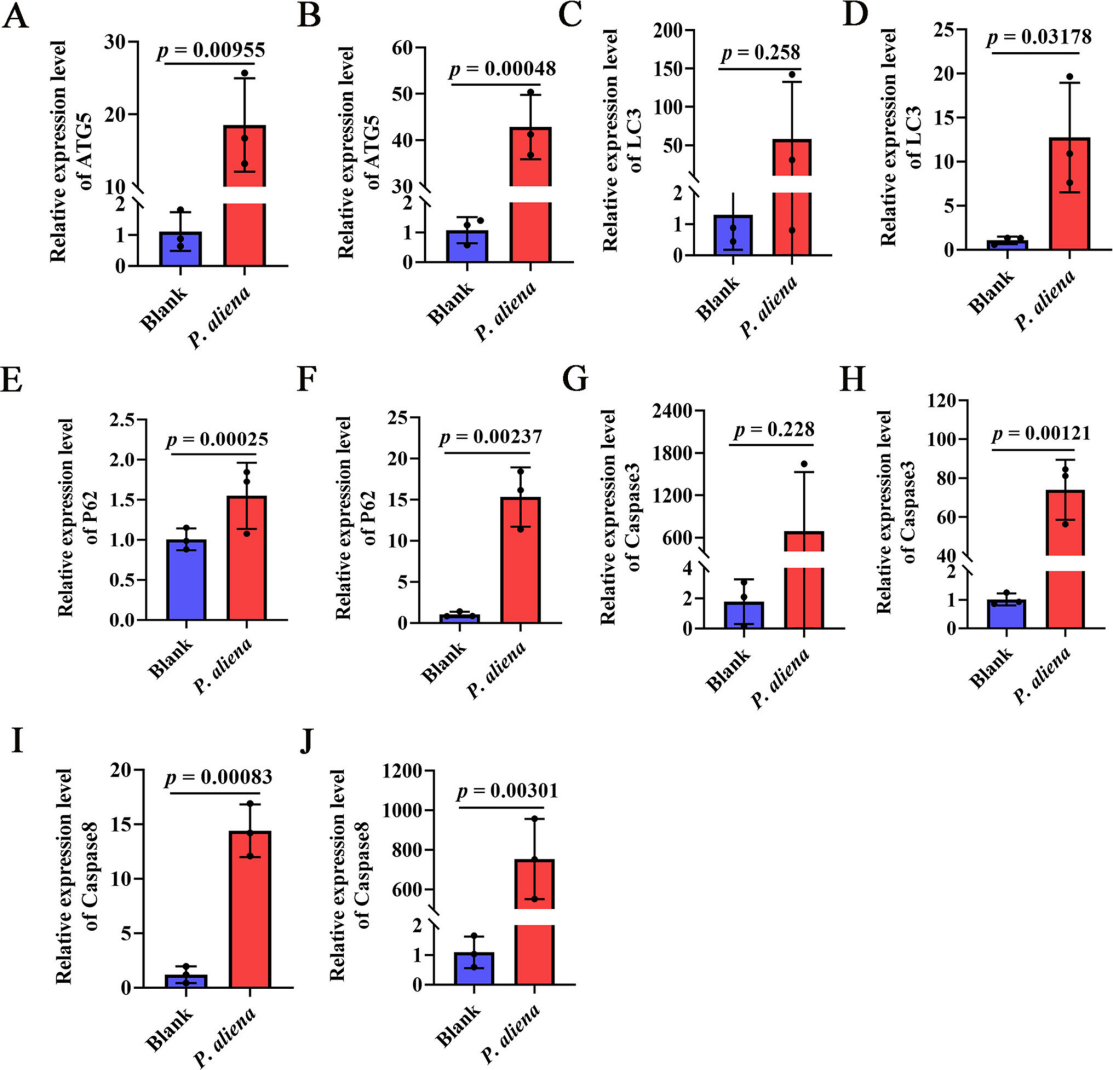
The relative mRNA expression levels of inflammatory programmed cell death-related genes in gills and mantle after *P. aliena* infection. (**A, C, E, G, and I**) The mRNA expression levels of *Cg*ATG5, *Cg*LC3, *Cg*P62, *Cg*Caspase3, and *Cg*Caspase8 in gills. (**B, D, F, H, and J**) The mRNA expression levels of *Cg*ATG5, *Cg*LC3, *Cg*P62, *Cg*Caspase3, and *Cg*Caspase8 in mantle. *P. aliena*: *P-1*. Error bars represent the mean ± S.D. (*N* = 3).

The expression levels of non-inflammatory programmed cell death-related genes in the gills and mantle were significantly higher after *P. aliena* infection. The mRNA expression levels of *Cg*ATG5 (Fig. 5A and B), *Cg*LC3 (Fig. 5D), *Cg*P62 (Fig. 5E and F), *Cg*Caspase3 (Fig. 5H), and *Cg*Caspase8 (Fig. 5I and J) after *P. aliena* infection were significantly increased, which were 10.39-fold, 40.03-fold, 12.01-fold, 1.53-fold, 14.85-fold, 73.03-fold, 12.06-fold, and 692.54-fold (*P* < 0.05) compared with those in the control group, respectively. There was no significant change in the mRNA expression levels of *Cg*LC3 in the gills (Fig. 5C) and *Cg*Caspase3 in the gills (Fig. 5G).

### The mRNA expression levels of inflammation-related factors in gills and mantle after *P. aliena* infection

**Fig 6:**
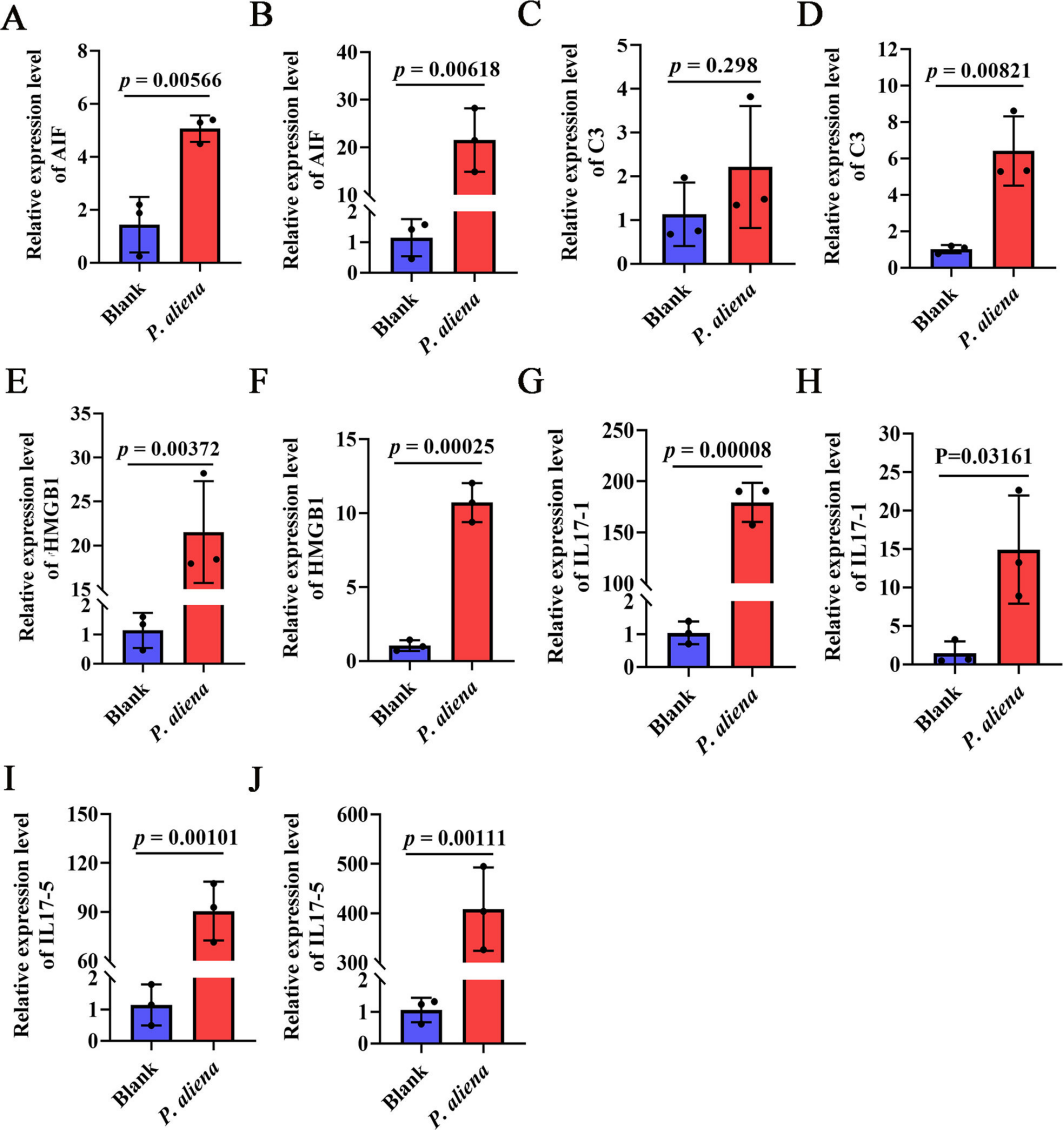
The relative mRNA expression levels of inflammation-related genes in gills and mantle after *P. aliena* infection. (**A, C, E, G, and I**) The mRNA expression levels of *Cg*AIF1, *Cg*C3, *Cg*HMGB1, *Cg*IL17-1, and *Cg*IL17-5 in gills. (**B, D, F, H, and J**) The mRNA expression levels of *Cg*AIF1, *Cg*C3, *Cg*HMGB1, *Cg*IL17-1, and *Cg*IL17-5 in mantle. *P. aliena*: *P-1*. Error bars represent the mean ± S.D. (*N* = 3).

The mRNA expression levels of inflammation-related genes in gills and mantle were significantly increased after *P. aliena* infection. In the challenged group, the mRNA expression levels of *Cg*AIF1 (Fig. 6A and B), *Cg*C3 (Fig. 6D), *Cg*HMGB1 (Fig. 6E and F), *Cg*IL17-1 (Fig. 6G and H), and *Cg*IL17-5 (Fig. 6I and J) increased significantly, which was 2.64-fold, 18.83-fold, 8.46-fold, 18.9-fold, 10.28-fold, 172.81-fold, 10.29-fold, 79.37-fold, and 387.14-fold of that in the control group (*P* < 0 0.05), respectively. No significant difference was observed in the mRNA expression level of *Cg*C3 in the gills (Fig. 6C).

### The bacterial community changes in the gills and mantle after *P. aliena* infection

**Fig 7:**
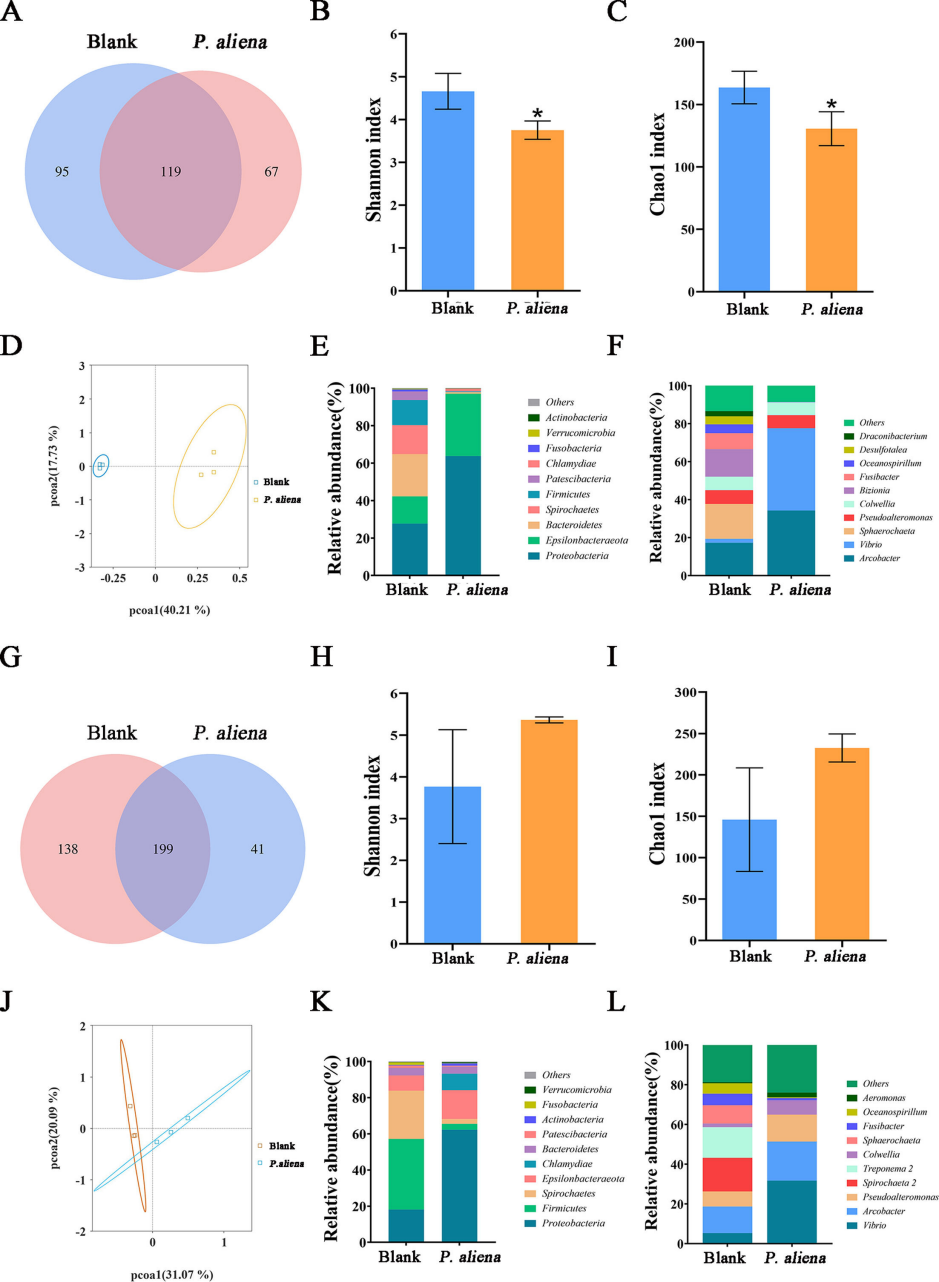
Analysis of the bacterial communities in gills and mantle after *P. aliena* infection. (**A and G**) Venn diagram analysis depicting the numbers of shared and unique ASVs among the control and challenged groups in the gills and mantle. (**B and H**) Alpha diversity of the Chao1 index comparisons in gills and mantle microbiota among the control and challenged groups. (**C and I**) Alpha diversity of Shannon index comparisons in gills and mantle microbiota among the control and challenged groups. (**D and J**) PCoA analysis of microbiota in gills and mantle among the control and challenged groups based on the Bray-Curtis distance metrics. (**E and K**) The composition of the microbiota in gills and mantle among the control and challenged groups at the phylum level. The top ten abundant phyla were shown, and the rest were indicated as “Others.” (**F and L**) Composition of microbiota in gills and mantle among the control and challenged groups at the genus level. The top ten abundant genera were shown, and the rest were indicated as “Others.”

In gills, the quality screening process yielded a total of 1,489,481 valid sequences, which were further clustered into 281 amplicon sequence variants (ASVs) between the two groups, while the control and challenged groups exhibited unique sets of 95 and 67 ASVs, respectively (Fig. 7A). Similarly, in mantle, there were 378 ASVs between the two groups, with an additional set of 138 and 41 unique ASVs found exclusively in the control and challenged groups, respectively (Fig. 7G). All samples displayed Good’s coverage indexes above 0.999, indicating sufficient sequencing depth. The alpha diversity of bacteria was assessed using the Chao1 index and the Shannon index. In gills, the control group exhibited significantly higher values compared to those of the challenged group (Fig. 7B and C). However, no significant difference was observed between the control group and the challenged group in the mantle (Fig. 7H and I). The principal coordinate analysis (PCoA), based on the Bray-Curtis distance metric, revealed a clear differentiation between microbial communities in gills and mantle in the two groups (Fig. 7D and J). This observation was further supported by Analysis of Similarities analysis (ANOSIM; R = 0.5185, *P* = 0.1).

At the phylum level, the microbial communities in gills were predominantly composed of Proteobacteria (27.72%–63.83%), Epsilonbacteraeota (14.51%–33.16%), Bacteroidetes (0.93%–22.59%), and Spirochaetes (0.02%–15.49%) (Fig. 7E). Similarly, at the phylum level, microbial communities in the mantle were dominated by Proteobacteria (18.13%–62.31%), Firmicutes (3.24%–39.06%), Spirochaetes (2.66%–26.69%), and Epsilonbacteraeota (8.44%–15.93%) (Fig. 7K). Compared with that in the control group, there was an increase in the relative abundance of Proteobacteria and Epsilonbacteraeota in the challenged group. At the genus level, *Vibrio* (2.13%–43.38%), *Arcobacter* (17.24%–34.26%) and *Pseudoalteromonas* (7.23%–6.83%) were identified as the most dominant microbiota in gills (Fig. 7F). Similarly, at the genus level, *Vibrio* (5.34%–31.71%), *Arcobacter* (13.34%–19.68%), and *Pseudoalteromonas* (7.63%–13.57%) were found to be the predominant microbiota in mantle (Fig. 7L). In comparison to that of the control group, there was an obvious increase in the relative abundance of *Vibrio*, *Arcobacter,* and *Pseudoalteromonas* in the challenged group.

## DISCUSSION

In recent years, the rapid development of the aquaculture industry, particularly the significant expansion in shellfish farming scale, has profoundly promoted coastal areas' economic growth (29). However, the frequent occurrence of bacterial diseases has emerged as a significant constraint on the robust advancement of shellfish aquaculture (3). At present, *Vibrio* has emerged as the predominant pathogen causing bacterial infections in shellfish (30). In our previous study, the potential pathogens *Pseudoalteromonas* and *Vibrio* were successfully isolated from diseased oysters. Importantly, one of the dominant strains was identified to be *P. aliena*. The genus *Pseudoalteromonas* is a novel genus that is different from the genera *Pseudoalteromonas* and *Alteromonas* (31). This bacterium was widely distributed in marine environments and was classified as a conditional pathogen for some aquatic species. However, there are still no reports regarding the pathogenicity of *Pseudoalteromonas* in oysters, and the role of this genus in pathogenesis has not been investigated in depth. In the present study, *P. aliena* was isolated and identified as a potential pathogen responsible for oysters, and its pathogenic mechanism was systematically clarified in oysters.

*Pseudoalteromonas* is a Gram-negative, ovoid, rod-shaped bacterium that moves by means of a single unsheathed polar flagellum. In the present study, *P. aliena* was a rod-shaped bacterium measuring approximately 1 µm in length, characterized by a single polar flagellum at one end. The number of *P. aliena* entered the logarithmic growth phase at 28°C following 2 h and achieved the stationary phase after 10 h. Hemolytic activity is regarded as one of the most critical characteristics for evaluating pathogenicity (32). The hemolytic activity of bacteria could result in the lysis of host red blood cells, induce local tissue damage and bacterial infection, and trigger a series of secondary reactions (33). In the present study, *P. aliena* exhibited strong hemolytic activity at 28°C, which might contribute to the elevated incidence of bacterial diseases during the summer. The results of the drug sensitivity test indicated that *P. aliena* exhibited high sensitivity to 17 different antibiotics, suggesting that its drug sensitivity was substantial, and these antibiotics might serve as effective agents for disease management.

The pathogenic bacteria challenged test is extensively employed to assess the virulence of pathogenic bacterial strains, particularly in research related to aquatic animal diseases and immunology (34). In the present study, immersion experiments were conducted to investigate the pathogenicity of *P. aliena* to oysters. During these experiments, the cumulative mortality rate reached 100% at 25°C by the ninth day. Histological analysis showed that tissue necrosis and pathological deposition of the organs were among the characteristics of bacterial infection. The exotoxin produced by *Vibrio* could induce cilia shedding and necrosis of epithelial cells in the gills of bivalves (35). In the present study, following infection with *P. aliena*, the gills were swollen and severely impaired, and the mantle emerged pustules, which presented symptoms analogous to those of wild diseased oysters. The gill filaments of infected oysters exhibited swelling and necrotic cells, and the mantle presented a loose histological structure with cavities and disruption of epithelial cells. These findings indicated that *P. aliena* represented a significant pathogen associated with disease in *C. gigas*.

ECP is a comprehensive term that encompasses various components produced and secreted extracellularly during the propagation and transmission of pathogenic bacteria. Proteases and toxins are pivotal in damaging host tissues and facilitating bacterial invasion and dissemination. A variety of enzymatic activities, including proteolytic enzymes, can be detected in the ECPs of pathogenic bacteria. Among these, proteases are pivotal in facilitating the pathogenic process. For example, in black sea bass (*Paralichthys olivaceus*), the ECPs of highly virulent and low-virulence strains of *Vibrio scophthalmi* exhibited LD_50_ values of 10.14 µg per fish and 15.99 µg per fish, respectively (36). In the present study, the ECPs from the *P. aliena* strain exhibited protease, amylase, and urease activities. Based on these findings, it could be reasonably inferred that the ECPs played a critical role in the pathogenicity of *P. aliena* toward oysters. The ECPs of *Pasteurella multocida* played a crucial role in the pathogenesis of pasteurellosis and exhibited high toxicity to fish (37). ECPs not only contributed to the process of bacterial invasion of the host but also possessed a certain capacity for host invasion. The ECPs of *V. aestuarianus* exhibited immunosuppressive effects on hemocytes of *C. gigas* (38). In the present study, at 25°C, the mortality rate of oysters rose conspicuously on the second day after the injection of various concentrations of ECPs, and the deceased oysters presented the same disease symptoms as those of wild diseased oysters. This indicated that the ECPs from *P. aliena* played a significant role as a contributing factor in the pathogenesis of shellfish diseases. In the present study, the ECPs were subjected to mass spectrometry detection, and a total of 214 proteins were identified. Among them, pathogenic proteins with a relatively high confidence level (CV ≥95%), including GroL, ClpB, and HtpG proteins, were identified. Among which, GroL was a chaperone protein that had been identified as a virulence protein in *Photorhabdus* and *Xenorhabdus* (39). ClpB was a crucial chaperone within the AAA+ protein family and played an essential role in bacterial survival under various environmental stresses, particularly heat shock, through its proteolytic activity (40). Furthermore, ClpB played a critical role in modulating the expression of virulence factors in several pathogenic bacteria (41). HtpG was a functional protein with pronounced ATPase activity that diminished the immune capacity of macrophages by impairing the host’s ability to activate them, thereby contributing to the delayed pathogenic process of *Edwardsiella tarda* in aquatic animals (42).

In the present study, according to the Gene Ontology annotation analysis of the identified proteins, these proteins primarily exhibited activities related to dehydratase, tRNA binding, unfolded protein response (UPR), ATP binding, and metal ion binding. The UPR was a cytoprotective mechanism employed to restore cellular homeostasis in the endoplasmic reticulum following physiological stress (43). Certain pathogens have evolved molecular mechanisms to enhance their survival and proliferation by either exploiting or inhibiting the UPR in host cells (44). In the pathogenic processes of bacteria, the metabolic regulatory network and the function and expression of virulence factors were interconnected; at the nexus of nutrient metabolism and virulence, one of the most significant nutritional factors was trace metal ions (45, 46). The ECPs of *P. aliena* also exhibited metal ion binding capabilities, and the reason might be that, to survive within the host, pathogenic bacteria could effectively utilize available nutrients from the surrounding environment. The above results suggested that ECPs, as the principal factor causing oyster diseases by *P. aliena*, mainly led to the occurrence of oyster diseases through the virulent proteins in ECPs.

Cell death plays an important role in restricting pathogenic bacterial infection (47). Currently, multiple modes of cell death, such as apoptosis (48), pyroptosis (49, 50), autophagy (51), and ferroptosis (52), have been discovered in shellfish. On the one hand, they played a significant role in resisting pathogen infections. However, excessive and dysregulated cell death might cause a strong inflammatory response in shellfish, thereby triggering individual death (53). *Cg*Ferritin, *Cg*GPX4, and *Cg*SLC40A1 from oysters were demonstrated to be associated with ferroptosis, an iron-dependent form of regulated cell death (54). *Cg*DLAT, *Cg*FDX1, and *Cg*SLC31A1 were associated with cuproptosis, a copper-driven type of cell death (55). *Cg*ATG5, *Cg*LC3, and *Cg*P62 played critical roles in autophagy (51). *Cg*Caspase8 and *Cg*Caspase3 were involved in the pyroptosis of oysters (56). In the present study, the mRNA expression levels of programmed cell death-related genes, such as *Cg*Ferritin, *Cg*GPX4, *Cg*SLC40A1, *Cg*DLAT, *Cg*FDX1, *Cg*SLC31A1, *Cg*ATG5, *Cg*LC3, *Cg*P62, *Cg*Caspase8, and *Cg*Caspase3, were significantly increased in gills and mantle after *P. aliena* infection. These results further implied that *P. aliena* mainly induced multiple types of cell death in oyster immune tissues and thereby caused the individual’s death.

Inflammation represents a defensive reaction of the organism to stimuli and plays a crucial role in resisting pathogen infections. Nevertheless, excessive inflammatory responses can result in tissue damage to the organism and, in serious cases, trigger individual death. Inflammatory factors refer to any elements capable of inducing tissue and cell damage, encompassing biological factors, physical factors, chemical factors, foreign substances, necrotic tissues, and allergic reactions (57). At present, inflammatory cytokines such as interleukin (IL), tumor necrosis factor, high mobility group box 1 (HMGB1), and allograft inflammatory factor-1 (AIF1) have been identified in shellfish (58–60). In the present study, the mRNA expression levels of *Cg*AIF1, *Cg*C3, *Cg*HMGB1, *Cg*IL17-1, and *Cg*IL17-5 were significantly increased in the gills and mantles infected with *P. aliena*. The obvious swelling and ciliary shedding were observed in the gills. These research findings suggested that *P. aliena* was able to induce inflammatory responses in oysters.

The microbiota of oysters was inherently dynamic and undergoes changes in response to stressors such as disease, antibiotics, and elevated temperatures (61). In the present study, the microflora of gills and mantles from both infected and healthy oysters was analyzed. The alpha diversity index reflected the richness and evenness of microbial communities. The control group in the gills demonstrated significantly higher values compared to the challenged group. In contrast, the alpha diversity of mantle microbiota in the infected group did not show significant changes. Beta diversity analysis revealed that infection with *P. aliena* markedly altered the microbial community structure of both gills and mantles in oysters. Bacterial infection may significantly alter the composition of the host microbial community (62). In the present study, the host microbial community underwent significant change across multiple taxonomic levels after *P. aliena* infection. At the phylum level, the relative abundance of Proteobacteria and Epsilonbacteraeota was significantly increased in the challenged group. Proteobacteria encompass a substantial number of opportunistic pathogens, and their presence might facilitate the activation of the host immune system and the maintenance of immune function (63). The relative abundance of Bacteroidetes and Firmicutes was significantly decreased in the challenged group. Many bacteria within the Firmicutes phylum could degrade complex carbohydrates and produce short-chain fatty acids, which positively influenced the immune responses (64). Additionally, lipopolysaccharides and flagellar proteins from the Bacteroidetes phylum could interact with host cell receptors, enhancing the host immune response via cytokine synthesis (65). These findings suggested that the decreased relative abundances of the Bacteroidetes and Firmicutes phyla might be linked to the immune responses in oysters. At the genus level, significant alterations in the composition of microbiota in the gill and mantle were observed, with increasing abundances of *Vibrio*, *Arcobacter,* and *Pseudoalteromonas* in the challenged group. *Vibrio* has also been identified as a potential pathogenic bacterium capable of causing disease in fish and shellfish (66–68). Investigations into bacterial community composition during summer mortality disease events had also revealed significant increases in the relative abundance of *Vibrio* (69, 70). Previous reports in *C. gigas* had demonstrated that higher abundances of *Arcobacter* functioned as opportunistic pathogens in hemolymph, and the increased densities of *Arcobacter* could be a contributing factor to host mortality. *Pseudoalteromonas* in affected oysters exhibited greater abundance compared to that of the unaffected oysters. These results indicated that *P. aliena* contributed to disease through synergistic interactions with *Vibrio* and *Arcobacter* in oysters.

In conclusion, the causative agent of a new emerging disease associated with massive mortality in farmed oysters was identified as *P. aliena. P. aliena* caused oyster disease mainly through the secretions of virulent proteins, including GroL, ClpB, and HtpG proteins. *P. aliena* could activate the host’s inflammatory and cell death-related immune responses and synergize with other pathogenic bacteria to induce morbidity in oysters. These results provided valuable insights for further elucidating the mechanisms underlying bacterial disease occurrence in oysters and developing effective strategies to prevent and control diseases caused by *Pseudoalteromonas* in oyster farming.

## Supplementary Material

Reviewer comments

## Data Availability

Data will be made available on request.

## SUPPLEMENTAL MATERIAL

OPEN PEER REVIEW reviewer-comments.pdf An accounting of the reviewer comments and feedback.

ASM does not own the copyrights to Supplemental Material that may be linked to, or accessed through, an article. The authors have granted ASM a non-exclusive, world-wide license to publish the Supplemental Material files. Please contact the corresponding author directly for reuse.
