# Supplementary material for: Isolation, identification, and pathogenicity of Pseudoalteromonas aliena associated with oyster disease outbreaks in summer
Source: Microbiol Spectr. 2025 Jun 23;13(8):e00173-25. doi: 10.1128/spectrum.00173-25 (PMC12323356; doi:10.1128/spectrum.00173-25)
Supplement: Reviewer comments [file reviewer-comments.pdf]

AMERICAN SOCIETY FOR MICROBIOLOGY | Microbiology Spectrum

# Isolation, identification and pathogenicity of Pseudoalteromonas aliena associated with oyster disease outbreaks in summer

Shuyi Mu, Jiejie Sun, Lei Gao, Yinan Li, Zihan Wang, Renle Chang, Jun Jiang, Liyan Wang, Lingling Wang, and Linsheng Song

*Corresponding Author(s): Jiejie Sun, Dalian Ocean University*



## Review Timeline:

| | |
|---|---|
| Submission Date: | January 16, 2025 |
| Editorial Decision: | April 26, 2025 |
| Revision Received: | May 15, 2025 |
| Accepted: | May 27, 2025 |

*Editor: Warish Ahmed*

*Reviewer(s): Disclosure of reviewer identity is with reference to reviewer comments included in decision letter(s). The following individuals involved in review of your submission have agreed to reveal their identity: Jialong Yang (Reviewer #1); Jiang-Feng Lan (Reviewer #2)*

## Transaction Report:

(Note: With the exception of the correction of typographical or spelling errors that could be a source of ambiguity, letters and reports are not edited. The original formatting of letters and referee reports may not be reflected in this compilation.)

**DOI: https://doi.org/10.1128/spectrum.00173-25**

Re: Spectrum00173-25 (Isolation, identification and pathogenicity of Pseudoalteromonas aliena associated with oyster disease outbreaks in summer)

Dear Prof. Jiejie Sun:

Thank you for the privilege of reviewing your work. Below you will find my comments, instructions from the Spectrum editorial office, and the reviewer comments.

Please return the manuscript within 60 days; if you cannot complete the modification within this time period, please contact me. If you do not wish to modify the manuscript and prefer to submit it to another journal, notify me immediately so that the manuscript may be formally withdrawn from consideration by Spectrum.

**Revision Guidelines**
To submit your modified manuscript, log into the submission site at https://spectrum.msubmit.net/cgi-bin/main.plex. Go to Author Tasks and click the appropriate manuscript title to begin. The information you entered when you first submitted the paper will be displayed; update this as necessary. Note the following requirements:

• Upload point-by-point responses to the issues raised by the reviewers in a file named "Response to Reviewers," NOT in your cover letter.
• Upload a compare copy of the manuscript (without figures) as a "Marked-Up Manuscript" file.
• Upload a clean .DOC/.DOCX version of the revised manuscript and remove the previous version.
• Each figure must be uploaded as a separate, editable, high-resolution file (TIFF or EPS preferred), and any multipanel figures must be assembled into one file.
• Any supplemental material intended for posting by ASM should be uploaded with their legends separate from the main manuscript. You can combine all supplemental material into one file (preferred) or split it into a maximum of 10 files with all associated legends included.

For complete guidelines on revision requirements, see our Submission and Review Process webpage. Submission of a paper that does not conform to guidelines may delay acceptance of your manuscript.

**Data availability:** ASM policy requires that data be available to the public upon online posting of the article, so please verify all links to sequence records, if present, and make sure that each number retrieves the full record of the data. If a new accession number is not linked or a link is broken, provide Spectrum production staff with the correct URL for the record. If the accession numbers for new data are not publicly accessible before the expected online posting of the article, publication may be delayed; please contact production staff (Spectrum@asmusa.org) immediately with the expected release date.

**Publication Fees:** For information on publication fees and which article types are subject to charges, visit our website. If your manuscript is accepted for publication and any fees apply, you will be contacted separately about payment during the production process; please follow the instructions in that e-mail. Arrangements for payment must be made before your article is published.

**ASM Membership:** Corresponding authors may join or renew ASM membership to obtain discounts on publication fees. Need to upgrade your membership level? Please contact Customer Service at Service@asmusa.org.

The ASM Journals program strives for constant improvement in our submission and publication process. Please tell us how we can improve your experience by taking this quick Author Survey.

Thank you for submitting your paper to Spectrum.

Sincerely,
Warish Ahmed
Editor
Microbiology Spectrum

Reviewer #1 (Comments for the Author):

The manuscript by Mu et al. demonstrated that P. aliena was the pathogenic bacterium for oysters and its pathogenicity mechanism was systematically clarified. This is a very systematic and in-depth work related to the pathogenic mechanism of pathogenic pathogens in oysters. There are still some comments and suggestions that need to be resolved before publication.
1. Line 125. For the drug sensitivity test, the criteria for categorizing sensitivity (S), intermediate (I), and resistance (R) are not

specified. Please clarify the standards used.
2. Were the enzyme activities of the extracellular products analyzed? If not, it is recommended to include such data, as it would strengthen the mechanistic insights of this study.
3. Line 56: The materials refer to 16s rDNA, whereas the keyword mentions16s rRNA.
4. Line 70-72. It is essential to incorporate appropriate references to substantiate the validity.
5. Line 247. Why was 16{degree sign}C chosen as the blank control group?
6. Line 168. Note the filter membranes with pore sizes of 0.45 mm and 0.22 mm.
7. Lines 178-180. The oysters are stated to be divided into five groups; however, only four groups are described subsequently. Additionally, please ensure adherence to writing conventions, specifically the proper use of spaces in concentration values.
8. Lines 233-235. In Figure 2, the F diagram is missing.
9. Lines 263-265: What is the rationale for specifically highlighting CgGPX4 in the discussion?
10. Please explicitly delineate the molecules associated with each type of cell death in the discussion section and provide a comprehensive explanation of the diverse mechanisms by which this bacterium induces cell death.
11. The author should provide a detailed explanation in the discussion section regarding the specific implications of changes in microbial communities, such as the biological and ecological significance of the reduced abundance of Firmicutes.
12. Does the P. aliena have been reported to exhibit pathogenicity to other species?
13. Why choose the oyster gills and mantle to investigate pathogenic mechanism of P. aliena?

Reviewer #2 (Comments for the Author):

This study investigated the pathogenicity of Pseudoalteromonas aliena on Crassostrea gigas and elucidated its pathogenicity mechanism using high-throughput sequencing and immune molecular expression profiling.
1. Line 147. Could you clarify why the soaking experiment employed a concentration of 1 × 105 CFU mL-1?
2. The results presented in Figure 5G were not statistically significant, whereas the findings in Figure 5H were statistically significant. Additionally, the data in Figure 5 was inconsistent with the expected fold-changes. Please ensure that Section 3.5 is thoroughly revised.
3. Line 229. The reported mortality rate results were inconsistent with the data presented in the chart.
4. Figure 3: The results section lacks a description of the findings presented in Fig. 3F.
5. Lines 66-67. For firstly introducing the specie names, please provide their full and complete designations.
6. Line 71. The description of the Pseudomonas genus lacks sufficient clarity.
7. Line 112. Note the description of "Zobell Marine Broth 2216E".
8. Lines 121-122. Please confirm that 100 μL bacterial suspension was inoculated into 100 mL 2216E liquid medium.
9. Line 143. Why does the sterile seawater rinse target cells rather than bacteria after centrifugation? Please verify.
10. Line 148: Why was the experimental group maintained at 25{degree sign}C?
11. Line 261. The results presented in Figure 4H do not demonstrate statistical significance. Please review and implement appropriate revisions.
12. In Figure 7, the label for P. aliena on the x-axis is presented in an unstandardized format without proper spacing between words.
13. How does the increased abundance of Vibrio, Arcobacter and Pseudoalteromonas contribute to oyster disease?
14. In the discussion section, it would be beneficial to provide more evidence about the connections between GroL, ClpB, or HtpG and the pathogenicity of marine organisms.
15. In the last part of the discussion, the guiding significance of this study for the subsequent prevention and control of shellfish industry diseases should be added.

Dear Prof. Warish Ahmed,

We would like to thank you and the reviewers for the comments and suggestions and for giving us a chance to revise our manuscript. We have considered all of them during the revision. The revised parts are marked with red color in the revised manuscript. Here is a summary of the revisions and responses.

Reviewer #1 (Comments for the Author):

The manuscript by Mu et al. demonstrated that *P. aliena* was the pathogenic bacterium for oysters and its pathogenicity mechanism was systematically clarified. This is a very systematic and in-depth work related to the pathogenic mechanism of pathogenic pathogens in oysters. There are still some comments and suggestions that need to be resolved before publication.

1. Line 125. For the drug sensitivity test, the criteria for categorizing sensitivity (S), intermediate (I), and resistance (R) are not specified. Please clarify the standards used. Response: Thanks for the reviewer's suggestions and they had been added in the revised MS (lines 130-133).

2. Were the enzyme activities of the extracellular products analyzed? If not, it is recommended to include such data, as it would strengthen the mechanistic insights of this study.

Response: Thanks for the reviewer's suggestions. The enzyme activities of the extracellular products were analyzed and related results were added in the revised MS (lines 32-33, 177-183, 246-248, 364-370).

3. Line 56: The materials refer to 16s rDNA, whereas the keyword mentions16s rRNA.

Response: We are sorry for our careless and they all have been corrected in the revised MS (lines 108, 215 and 235).

4. Line 70-72. It is essential to incorporate appropriate references to substantiate the validity.

Response: We are sorry for our careless and it has been added in the revised MS (line 69).

5. Line 247. Why was 16°C chosen as the blank control group?

Response: Thanks for the reviewer's comments. It has been demonstrated that 16°C represents the optimal and normal growth temperature for oysters. Therefore, this temperature was chosen as the blank control group in the experiment.

6. Line 168. Note the filter membranes with pore sizes of 0.45 mm and 0.22 mm.

Response: We are sorry for our careless and they have been corrected in the revised MS (line 169).

7. Lines 178-180. The oysters are stated to be divided into five groups; however, only four groups are described subsequently. Additionally, please ensure adherence to writing conventions, specifically the proper use of spaces in concentration values.

Response: Thanks for the reviewer's suggestions and they had been corrected in the revised MS (lines 187-190).

8. Lines 233-235. In Figure 2, the F diagram is missing.

Response: Thanks and it had been added in the revised MS (line 240).

9. Lines 263-265: What is the rationale for specifically highlighting *Cg*GPX4 in the discussion?

Response: We are sorry for our careless and it has been corrected in the revised MS (line 269).

10. Please explicitly delineate the molecules associated with each type of cell death in the discussion section and provide a comprehensive explanation of the diverse mechanisms by which this bacterium induces cell death.

Response: Thanks for the reviewer's suggestions and they had been added in the revised MS (lines 402-407).

11. The author should provide a detailed explanation in the discussion section regarding the specific implications of changes in microbial communities, such as the biological and ecological significance of the reduced abundance of Firmicutes.

Response: Thanks for the reviewer's suggestions and they had been added in the revised MS

(lines 436-442).

12. Does the *P. aliena* have been reported to exhibit pathogenicity to other species?

Response: Thank you for the reviewer's comments. *Pseudoalteromonas* species were repoted to be pathogenic to marine organisms, such as fish [1, 2], crab [3], abalone [4], and alga [5]. While no reports are regarding the pathogenicity of *P. aliena* to other species.

13. Why choose the oyster gills and mantle to investigate pathogenic mechanism of *P. aliena*?

Response: Thank you for the reviewer's comments. The selection of oyster gills and mantle was based on their status as primary infection sites for *P. aliena*, evidenced by distinct histopathological lesions. For example, the gills displayed epithelial hyperplasia, filament fusion, and necrotic foci with hemolymph coagulates, while the mantle exhibited greenish discoloration, pustules, and structural disintegration.

Reviewer #2 (Comments for the Author):

This study investigated the pathogenicity of *Pseudoalteromonas aliena* on *Crassostrea gigas* and elucidated its pathogenicity mechanism using high-throughput sequencing and immune molecular expression profiling.

1. Line 147. Could you clarify why the soaking experiment employed a concentration of $1 \times 10^5$ CFU $mL^{-1}$?

Response: Thanks for the reviewer's suggestions. The bacterial strain *P. aliena* was adjusted to a concentration of $1\times10^8$ CFU $mL^{-1}$. In the immersion experiment, 10 mL of this bacterial suspension was added to 10 L of seawater, resulting in a final concentration of *P. aliena* at $1\times10^5$ CFU $mL^{-1}$ in the experimental setup. Additionally, in aquatic animal immersion experiments, bacterial suspensions are typically used at concentrations around $1\times10^5$ CFU $mL^{-1}$. Therefore, we adopted this concentration for our study.

2. The results presented in Figure 5G were not statistically significant, whereas the findings in Figure 5H were statistically significant. Additionally, the data in Figure 5 was inconsistent with the expected fold-changes. Please ensure that Section 3.5 is thoroughly revised.

Response: Thanks for the reviewer's comments and suggestions. They all had been corrected in the revised MS (lines 277-280).

3. Line 229. The reported mortality rate results were inconsistent with the data presented in the chart.

Response: Thanks for the reviewer's comments and suggestions. It had been corrected in the revised MS (line 233).

4. Figure 3: The results section lacks a description of the findings presented in Fig. 3F.

Response: Thanks for the reviewer's comments and it had been added in the revised MS (line 254).

5. Lines 66-67. For firstly introducing the specie names, please provide their full and complete designations.

Response: Thanks for the reviewer's suggestions and it had been added in the revised MS (lines 65-67).

6. Line 71. The description of the Pseudomonas genus lacks sufficient clarity.

Response: Thanks for the reviewer's comments and it had been added in the revised MS (lines 70-71).

7. Line 112. Note the description of "Zobell Marine Broth 2216E".

Response: Thanks for the reviewer's suggestions and it had been corrected in the revised MS (lines 100-101 and 152).

8. Lines 121-122. Please confirm that 100 μL bacterial suspension was inoculated into 100 mL 2216E liquid medium.

Response: We are sorry for our careless and it has been corrected the revised MS (line 121).

9. Line 143. Why does the sterile seawater rinse target cells rather than bacteria after centrifugation? Please verify.

Response: We are sorry for our careless and it has been corrected in the revised MS (line 143).

10. Line 148: Why was the experimental group maintained at 25°C?

Response: Thanks for the reviewer's suggestions. *P. aliena* was originally isolated from diseased oysters during summer when the ambient water temperature averaged 25 °C (measured during pathogen sampling). This temperature setting ensures ecological relevance by mimicking natural infection conditions.

11. Line 261. The results presented in Figure 4H do not demonstrate statistical significance. Please review and implement appropriate revisions.

Response: Thanks for the reviewer's suggestions and it had been corrected in the revised MS (line 269).

12. In Figure 7, the label for *P. aliena* on the x-axis is presented in an unstandardized format without proper spacing between words.

Response: Thanks for the reviewer's suggestions. The images all had been improved in figure 7.

13. How does the increased abundance of *Vibrio*, *Arcobacter* and *Pseudoalteromonas* contribute to oyster disease?

Response: Thanks for the reviewer's comments. At the genus level, the microbial communities in oyster gills and mantle showed significant compositional shifts, with *Vibrio*, *Arcobacter* and *Pseudoalteromonas* exhibiting markedly increased abundance in challenged groups. *Vibrio* and Arcobacter were recognized as potential pathogens linked to diseases in fish and shellfish, and their proliferation likely contributed to host mortality [6-8]. Furthermore, *P. aliena* might have synergized with *Vibrio* and *Arcobacter* through microbial interactions to exacerbate disease progression.

14. In the discussion section, it would be beneficial to provide more evidence about the connections between GroL, ClpB, or HtpG and the pathogenicity of marine organisms.

Response: Thanks for the reviewer's suggestions and it had been added in the revised MS (lines 381-382).

15. In the last part of the discussion, the guiding significance of this study for the subsequent prevention and control of shellfish industry diseases should be added.

Response: Thanks for the reviewer’s suggestions and it had been added in the revised MS (lines 459-461).

We did our best to address all of reviewers’ suggestions and comments during the revision. Hoping the revised MS is acceptable for publication. We are looking forward to hearing your decision. Thank you very much for your help.

Best wishes.

Yours Sincerely

Jiejie Sun

References:

[1] Nelson, E.J., Ghiorse, W.C., 2002. Isolation and identification of *Pseudoalteromonas piscicida* strain Cura-d associated with diseased damselfish (*Pomacentridae*) eggs. J. Fish Dis 22, 253-260. https://doi.org/10.1046/j.1365-2761.1999.00168.x

[2] Pujalte, M.J., Sitjà-Bobadilla, A., Macián, M.C., Álvarez-Pellitero, P., Garay, E., 2007. Occurrence and virulence of *Pseudoalteromonas* spp. in cultured gilthead sea bream (Sparus aurata L.) and European sea bass (Dicentrarchus labrax L.). Molecular and phenotypic characterisation of *P. undina* strain U58. Aquaculture 271(1), 47-53. https://doi.org/https://doi.org/10.1016/j.aquaculture.2007.06.015

[3] Memon, A.J.J.J.o.A., Advances, V., 2011. Pathogenicity and Antibiotic Sensitivity of Pathogenic Flora Associated with the Gut of Blue Swimming Crab, *Portunus pelagicus* (Linnaeus, 1857). Trop Life Sci Res. 2014 Aug;25(1):13–27. http://dx.doi.org/10.3923/javaa.2011.2106.2119

[4] Li, M., Wu, W., You, W., Huang, S., Huang, M., Luo, X., Lu, Y., Ke, C., Xie, Q.J.A., 2021. A novel screening method for the detection of *Pseudoalteromonas shioyasakiensis*, an emerging opportunistic pathogen that caused the mass mortality of juvenile Pacific abalone (*Haliotis discus hannai*) during a record-breaking heat wave. J. Environ. Sci.545 (2021) 737191 http://dx.doi.org/10.1016/j.aquaculture.2021.737191.

[5] Sawabe, T., Tanaka, R., Iqbal, M.M., Tajima, K., Ezura, Y., Ivanova, E.P., Christen, R., 2000. Assignment of Alteromonas elyakovii KMM 162T and five strains isolated from spot-wounded fronds of Laminaria japonica to *Pseudoalteromonas elyakovii* comb. nov. and the extended description of the species. Int. j. syst. bacteriol 50 Pt 1, 265-271. https://doi.org/10.1099/00207713-50-1-265

[6] Green, T.J., Nachshon, S., King, W.L., Maurizio, L., Seymour, J.R., David, R.J.M.E., 2018. Simulated Marine Heat Wave Alters Abundance and Structure of *Vibrio Populations* Associated with the *Pacific Oyster* Resulting in a Mass Mortality Event. Microb Ecol. 2019 Apr;77(3):736-747. https://doi.org/10.1007/s00248-018-1242-9

[7] Mendoza, M., GÃÂ¼iza, L., Martinez, X., Caraballo, X., Rojas, J., Aranguren, L.F., Salazar, M., 2013. A novel agent (*Endozoicomonas elysicola*) responsible for epitheliocystis in cobia *Rachycentrum canadum larvae*. Dis. Aquat. Org. 2013 Sep 24;106(1):31-7. https://doi.org/10.3354/dao02636

[8] Qian, H., Zhang, M., Liu, G., Lu, T., Sun, L., Pan, X., 2019. Effects of different concentrations of *Microcystis aeruginosa* on the intestinal microbiota and immunity of zebrafish (*Danio rerio*). Chemosphere 214, 579-586. https://doi.org/10.1016/j.chemosphere.2018.09.156

Re: Spectrum00173-25R1 (Isolation, identification and pathogenicity of Pseudoalteromonas aliena associated with oyster disease outbreaks in summer)

Dear Prof. Jiejie Sun:

Your manuscript has been accepted, and I am forwarding it to the ASM production staff for publication. Your paper will first be checked to make sure all elements meet the technical requirements. ASM staff will contact you if anything needs to be revised before copyediting and production can begin. Otherwise, you will be notified when your proofs are ready to be viewed.

**Data Availability:** ASM policy requires that data be available to the public upon online posting of the article, so please verify all links to sequence records, if present, and make sure that each number retrieves the full record of the data. If a new accession number is not linked or a link is broken, provide production staff with the correct URL for the record. If the accession numbers for new data are not publicly accessible before the expected online posting of the article, publication may be delayed; please contact ASM production staff immediately with the expected release date.

**Publication Fees:** For information on publication fees and which article types have charges, please visit our website. We have partnered with Copyright Clearance Center (CCC) to collect author charges. If fees apply to your paper, you will receive a message from no-reply@copyright.com with further instructions. For questions related to paying charges through RightsLink, please contact CCC at ASM_Support@copyright.com or toll free at +1-877-622-5543. CCC makes every attempt to respond to all emails within 24 hours.

**ASM Membership:** Corresponding authors may join or renew ASM membership to obtain discounts on publication fees. Need to upgrade your membership level? Please contact Customer Service at Service@asmusa.org.

**PubMed Central:** ASM deposits all Spectrum articles in PubMed Central and international PubMed Central-like repositories immediately after publication. Thus, your article is automatically in compliance with the NIH access mandate. If your work was supported by a funding agency that has public access requirements like those of the NIH (e.g., the Wellcome Trust), you may post your article in a similar public access site, but we ask that you specify that the release date be no earlier than the date of publication on the Spectrum website.

**Embargo Policy:** A press release may be issued as soon as the manuscript is posted on the Spectrum Latest Articles webpage. The corresponding author will receive an email with the subject line "ASM Journals Author Services Notification" when the article is available online.

The ASM Journals program strives for constant improvement in our submission and publication process. Please tell us how we can improve your experience by taking this quick Author Survey.

Thank you for submitting your paper to Spectrum.

Sincerely,
Warish Ahmed
Editor
Microbiology Spectrum

Reviewer #1 (Comments for the Author):

The authors have addressed all my concerns.

Reviewer #2 (Comments for the Author):

The author has corrected all my questions.
